# Rational Manipulation of mRNA Folding Free Energy Allows Rheostat Control of Pneumolysin Production by *Streptococcus pneumoniae*

**DOI:** 10.1371/journal.pone.0119823

**Published:** 2015-03-23

**Authors:** Fábio E. Amaral, Dane Parker, Tara M. Randis, Ritwij Kulkarni, Alice S. Prince, Mimi M. Shirasu-Hiza, Adam J. Ratner

**Affiliations:** 1 Department of Pediatrics, Columbia University, New York, NY United States of America; 2 Department of Pharmacology, Columbia University, New York, NY, United States of America; 3 Department of Genetics & Development, Columbia University, New York, NY, United States of America; 4 Life and Health Sciences Research Institute (ICVS), School of Health Sciences, University of Minho, Braga, Portugal; 5 ICVS/3B's, PT Government Associate Laboratory, Braga/Guimarães, Portugal; University of Kansas Medical Center, UNITED STATES

## Abstract

The contribution of specific factors to bacterial virulence is generally investigated through creation of genetic “knockouts” that are then compared to wild-type strains or complemented mutants. This paradigm is useful to understand the effect of presence vs. absence of a specific gene product but cannot account for concentration-dependent effects, such as may occur with some bacterial toxins. In order to assess threshold and dose-response effects of virulence factors, robust systems for tunable expression are required. Recent evidence suggests that the folding free energy (ΔG) of the 5’ end of mRNA transcripts can have a significant effect on translation efficiency and overall protein abundance. Here we demonstrate that rational alteration of 5’ mRNA folding free energy by introduction of synonymous mutations allows for predictable changes in pneumolysin (PLY) expression by *Streptococcus pneumoniae* without the need for chemical inducers or heterologous promoters. We created a panel of isogenic *S*. *pneumoniae* strains, differing only in synonymous (silent) mutations at the 5’ end of the PLY mRNA that are predicted to alter ΔG. Such manipulation allows rheostat-like control of PLY production and alters the cytotoxicity of whole *S*. *pneumoniae* on primary and immortalized human cells. These studies provide proof-of-principle for further investigation of mRNA ΔG manipulation as a tool in studies of bacterial pathogenesis.

## Introduction

Delineating specific bacterial factors involved in interaction with the host is crucial to understanding mechanisms of pathogenesis and developing targeted therapies. Classically, such investigations involve construction of bacteria with disruptions in the genes encoding candidate factors (“knockouts”; KO) and comparison with wild-type (WT) or complemented strains. However, the level of expression of bacterial genes may also play a role in pathogenesis. The KO vs. WT paradigm cannot account for such differences, and although systems exist for inducible expression of specific bacterial genes, these generally rely on exogenous activators such as tetracycline that may have variable delivery to relevant sites during infection [1]. Thus, it is desirable to develop strategies for genetically encoded, tunable, rheostat-like control of bacterial gene expression.

A number of factors determine the efficiency of protein production. As the genetic code is degenerate, choice among synonymous codons may play a role in translational efficiency [2]. Early models postulated a direct relationship between tRNA availability and protein production, implying that use of non-optimal codons (i.e. those that are rarely used among other genes in the species being studied) would lead to decreased protein production on the basis of tRNA scarcity [2–4]. Codon-optimization has been used as a means to increase production of heterologous genes in *E*. *coli* and other host species [5]. Conversely, deoptimization of codons or codon pairs has been employed to rationally decrease protein production from natively transcribed genes [6,7]. Kudla et al. examined a library of synonymous codon substitutions in heterologously expressed green fluorescent protein (GFP) in *E*. *coli* and demonstrated that the mRNA folding free energy (ΔG), particularly at the 5’ terminus, correlates strongly with translational efficiency and with overall protein production [8]. Goodman et al. investigated the role of both codon bias and mRNA ΔG in synthetic reporters and confirmed a prominent role for ΔG in shaping expression levels of individual genes [9].

We used *Streptococcus pneumoniae* (pneumococcus), a major human pathogen with robust tools for genetic manipulation [10], as a model organism. We constructed a panel of isogenic pneumococcal strains differing only in synonymous codons at the 5’ end of the gene encoding pneumolysin (PLY), an established virulence factor [11,12], without antibiotic selection cassettes or exogenous promoters. These modifications altered the predicted mRNA ΔG and resulted in graded PLY production, thus affecting host-bacterial interactions and providing proof-of-principle for the use of rational modification of mRNA ΔG as a means to control protein production.

## Materials and Methods

### Ethics statement

The use of primary human erythrocytes following written informed consent was approved by the Institutional Review Board of Columbia University Medical Center. The human cell line A549 was obtained from ATCC (catalog number CCL-185).

### Calculation of ΔG and codon adaptation index

Sequence folding free energies were calculated using the DINAMelt/Quikfold web server (http://mfold.rna.albany.edu/?q=DINAMelt/Quickfold) [13]. The software uses predicted free energies at 37°C and enthalpies from the Turner laboratory at the University of Rochester in Rochester, NY. For ΔG calculations we used version 3.0 free energies. Codon adaptation index (CAI) based on the set of highly expressed genes from *Streptococcus pneumoniae* strain D39 was calculated using the CAIcal server (http://genomes.urv.es/CAIcal/) [14].

### Bacterial strains, primers, plasmids, transformation and growth conditions

**Fig 1:**
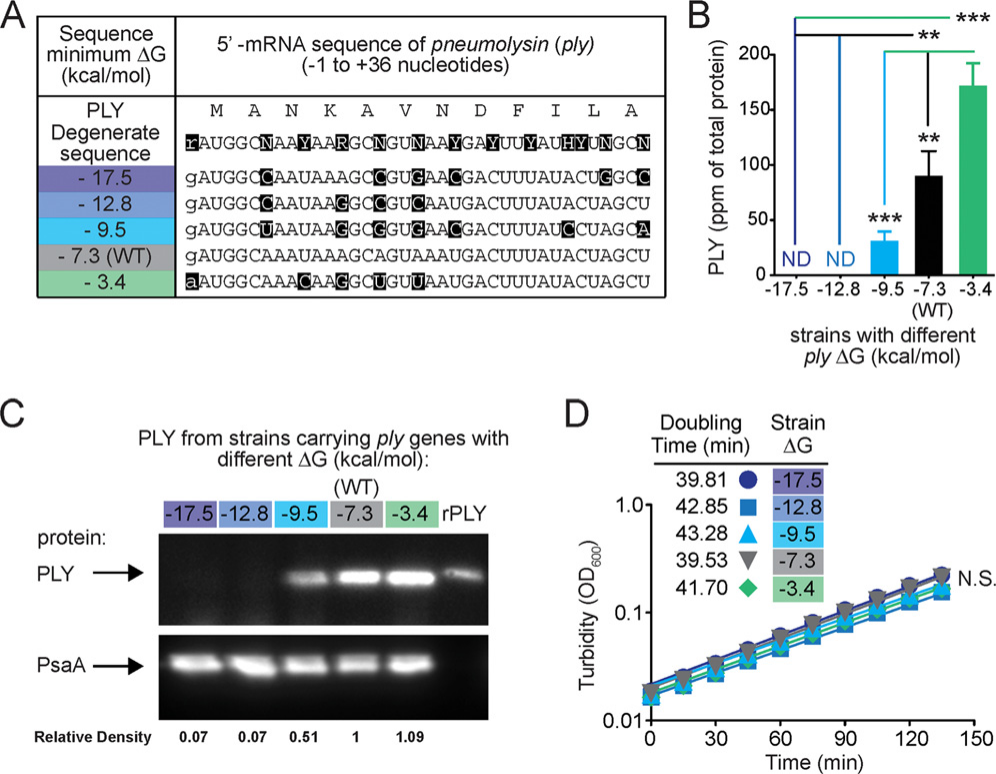
Production of PLY increases with increasing *ply* 5’ mRNA folding free energy. (A) Summary of the silent mutations engineered in the 5’-mRNA region of the *ply* gene. IUPAC Nucleic Acid Codes: Y (pYrimidine); R (puRine); N (aNy); H (not G). (B) Increasing levels of predicted *ply* 5’ mRNA ΔG correlate with increased PLY production as determined by ELISA normalized to total pneumococcal protein. Data are shown as mean of 4 experiments ± SEM. *, *P* < 0.05; **, *P* < 0.01; ***, *P* < 0.001 (ANOVA). (C) Western blot (top, anti-PLY; bottom, anti-PsaA loading control) demonstrates different levels of PLY production in engineered strains (lanes 1–5) compared to recombinant PLY (lane 6) as a control. Numbers beneath each lane indicate the ratio of PLY to PsaA band intensity, normalized to the value for the wild-type (ΔG = -7.3) strain in lane 4. (D) Measurement of turbidity (OD_600_) and calculated doubling times shows identical growth kinetics among isogenic *S*. *pneumoniae* strains with altered *ply* 5’ sequences. (N.S. = not significant; ANOVA)

We used the Janus cassette [10], to create a panel of isogenic *S*. *pneumoniae* R6 derivatives with a wide range of ΔG values (-17.5 to -3.4 kcal/mol) for the 5’ end of *ply* gene (-4 to +38 bp, with 1 being the A in the first ATG). To control for unexpected effects of transformations we recreated, and sequenced, a strain with the WT *ply* allele for comparison. Each of the new *ply* constructs was inserted into the native chromosomal locus, without resistance markers or alteration in predicted primary amino acid sequence. All transformed loci were confirmed by sequencing. All constructs were first assembled in *E*. *coli* TOP10 using plasmid pCR2.1-TOPO (Invitrogen), amplified by PCR, and transformed into pneumococcus. Forward primers differed in nucleotides encoding the initial PLY region according to Fig. 1, consistent with the following degenerate sequence: 5’-GAAR **ATG** GCN AAY AAR GCN GTN AAY GAY TTY ATH YTN GCN ATG AAT TAC G-3’ (start codon in bold), and the reverse primer was 5’-CTA GTC ATT TTC TAC CTT ATC TTC T-3’. Constructs for pneumococcal transformation were assembled by overlap-extension PCR and included 570 bp upstream of *ply* (forward primer 5’-GGT TAT TGG CGA CAA GCA TT-3’) and 769 bp downstream (reverse primer 5’-CCT GCT AAG ATG GTC TTG CC-3’).

Briefly, pneumococcal transformation was achieved with use of semisynthetic casein plus yeast extract (C+Y) medium [15]. Cultures were grown at 37°C in C+Y pH 6.8 to 0.15 OD_600_, then diluted 1:20 fold into C+Y pH 8.0 (total volume 1 mL) at 30°C, supplemented with 1 μg of CSP-1 and 11 μL of 1% CaCl_2_ and incubated for 12 minutes. After adding 1 μg DNA, cultures were incubated for 40 minutes at 30°C, followed by 90 minutes at 37°C, followed by appropriate selection in agar plates incubated overnight at 37°C, 5% CO_2_.

*E*. *coli* were grown in lysogeny broth (LB) or agar supplemented with appropriate antibiotics for plasmid selection (ampicillin 200 μg/mL or kanamycin 50 μg/mL). *S*. *pneumoniae* were grown in tryptic soy broth (TSB) or agar supplemented with 200 U/mL of catalase (Worthington Biochemical), and appropriate antibiotics for strain selection (streptomycin 150 μg/mL or kanamycin 200 μg/mL).

### Western blotting and ELISA

Protein separation, transfer, and immunoblotting were performed as previously described [16]. In blots, PLY was detected using 1:1000 dilution of mouse monoclonal anti-pneumolysin (1F11), PsaA was detected using 1:25000 dilution mouse monoclonal PsaA antibody (8G12) [17]. Densitometry was performed using the gel analysis plugin implemented in ImageJ (version 1.45s; National Institutes of Health). PLY was detected by indirect ELISA using monoclonal anti-pneumolysin (1F11; 1:1000 dilution).

### qRT-PCR and mRNA half-life

*S*. *pneumoniae* were grown to early exponential stage (OD_600_ = 0.3) and stored in RNAlater (Ambion). RNA was isolated using the RiboPure-Bacteria Kit (Ambion) with DNase treatment. cDNA was made using the SuperScript III RT Kit (Invitrogen), qRT-PCR was performed using Quanta SYBR Green PCR Master Mix in a StepOne Plus thermal cycler (Applied Biosystems).

The primers for the *ply* gene of *S*. *pneumoniae* (GenBank accession no. M17717) for real-time reverse-transcription PCR were previously described [18]. Samples were normalized to *S*. *pneumoniae* 16S rRNA detected with forward (5’-GCC TAC ATG AAG TCG GAA TCG-3’) and reverse (5’-TAC AAG GCC CGG GAA CGT-3’) primers. In separate experiments, transcription was arrested by addition of rifampicin to 10 μg/mL and mRNA abundance and decay were assessed by qRT-PCR of serially collected samples normalized to the 30 sec time point. mRNA half-life was calculated from best-fit exponential decay curves.

### Hemolysis assay

Primary human erythrocytes (0.5% final concentration) were incubated for 30 min at 37°C with an equal volume of heat-killed (52°C, 20 min) log-phase (OD_600_ = 0.25) *S*. *pneumoniae* R6 strains suspended in PBS. Intact erythrocytes were pelleted by centrifugation, and hemolysis was measured by assaying hemoglobin concentration in the supernatant (OD415 of a 100μl sample). Hemolysis values were normalized to 0% (vehicle control) and 100% lysis (1% Triton X-100) controls as previously described [16,19].

### Cell culture and cytotoxicity assay

A549 respiratory epithelial cells (obtained from ATCC, catalog number CCL-185) were grown in minimum essential medium (MEM) with 10% fetal bovine serum as described [20]. Cell monolayers (80–90% confluent in sterile 24-well plates) were incubated in MEM without serum overnight prior to stimulation with ~10^8^ cfu of the indicated *S*. *pneumoniae* strains for 12 hrs. LDH cytotoxicity assay (Roche) was performed as per the manufacturer’s instructions, and values were normalized to 0% (vehicle control) and 100% lysis (1% Triton X-100) controls as previously described [21].

### Statistical analysis

Statistics were performed with Prism 5 software (GraphPad, Inc.). Data were analyzed by analysis of variance followed by post-tests to account for multiple comparisons, as appropriate. In vitro experiments were performed at least three times with at least three technical replicates. Bars represent SEM. Values of *P* ≤ 0.05 were considered significant.

## Results

### Production of PLY increases with increasing ΔG

Using a degenerate nucleotide sequence derived from the *S*. *pneumoniae* R6 PLY protein sequence, we designed several candidate 5’ mRNA regions for the *ply* gene (1416 nucleotides total) that differed in predicted minimum mRNA folding ΔG (calculated using the standard mfold algorithm over the region from-4 to +38 nucleotides [13]). Each of these genes only contained synonymous mutations, leading to the production of identical amino acid sequences, but covering a wide range of predicted ΔG values. Because only a small number of codons was altered in each strain (4–7 codons/strain), the CAI for the full length *ply* gene based on highly expressed genes of *S*. *pneumoniae* D39 was unchanged (~0.38 in all strains). The two strains with the lowest 5’ mRNA ΔG (-17.5 and -12.8) also had lower CAI over the first 39 bp (0.227 and 0.236, respectively) than the other three strains (0.404, 0.365, and 0.375 for the ΔG = -9.5, -7.3, and -3.4 strains, respectively). The predicted ΔG of the native *ply* 5’ mRNA is-7.3 kcal/mol, and this strain was included as a control in all experiments (Fig. 1A). These alterations resulted in differential expression of PLY protein. With increasing ΔG values, we observed higher PLY levels as measured by both ELISA (Fig. 1B) and western blot (Fig. 1C). As an important control for changes in bacterial growth that would obviously impact total protein levels, we confirmed that all strains exhibited similar growth rates and doubling times (Fig. 1D). Thus alterations of the *ply* DNA sequence that alter the predicted ΔG of its resulting mRNA led to predictable and quantitative alterations in translated protein levels.

### Translation kinetics drive the relationship between ΔG and protein abundance

**Fig 2:**
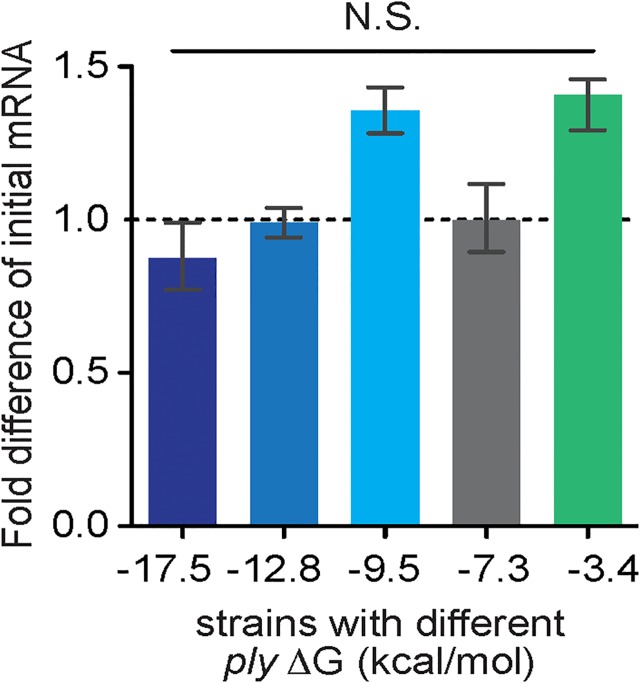
Engineered differences in 5’ mRNA ΔG do not alter *ply* mRNA quantity. Fold difference in *ply* mRNA levels (y axis) was calculated relative to wild-type (ΔG = -7.3) at early exponential phase. Relative mRNA amounts were calculated by ΔΔC_T_ normalized to pneumococcal 16S rRNA. (N.S. = not significant; ANOVA)

In order to understand the mechanisms underlying the relationship between mRNA ΔG and protein production in *S*. *pneumoniae*, we tested whether differences in *ply* mRNA levels correlated with predicted ΔG values for the 5’ mRNA and could account for differences in PLY protein levels. We used real-time reverse transcriptase PCR to quantify *ply* mRNA normalized to 16S rRNA for each strain. There were no statistically significant differences between wild-type and engineered strains in mRNA abundance (Fig. 2). Thus the absolute levels of mRNA abundance do not correlate with predicted ΔG values or with differences in PLY protein levels.

**Fig 3:**
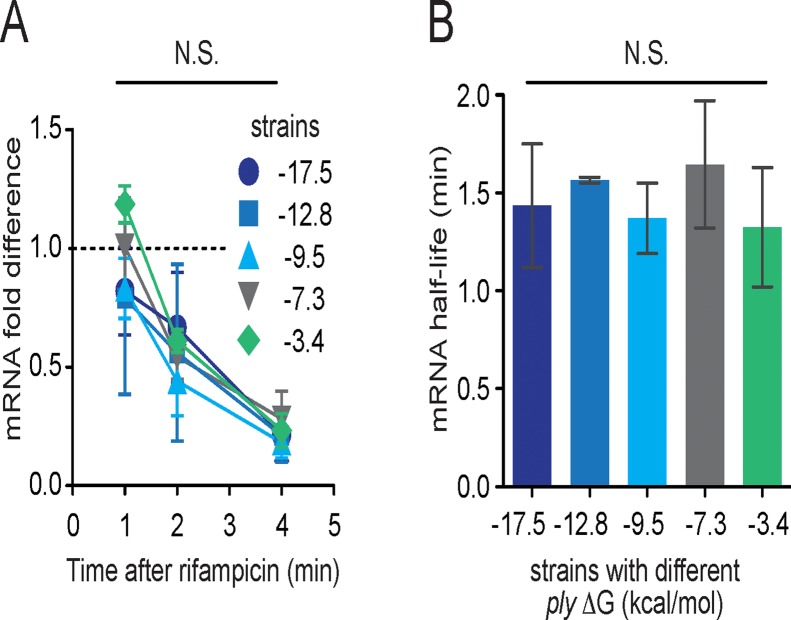
Differences in PLY production do not correlate with mRNA decay kinetics. (A) Abundance of *ply* mRNA following transcriptional arrest with rifampicin. mRNA quantities were measured by qRT-PCR and normalized to 16S rRNA. (N.S. = not significant; ANOVA). (B) Differences in *ply* 5’ mRNA ΔG do not alter mRNA half-life values, as calculated from best-fit exponential decay curves from data in (A). (N.S. = not significant; ANOVA)

Decay kinetics of mRNA can also alter overall protein production. To test whether altered decay kinetics correlated with differences in PLY protein levels, we next assessed mRNA decay at several time points following arrest of transcription with rifampicin (Fig. 3A). Decay curves were similar and did not differ statistically. We also calculated mRNA half-lives based on mRNA abundance following transcription arrest. mRNA half-lives for each *ply* sequence were all approximately 1.5 min, and there were no statistically significant differences among groups (Fig. 3B). Taken together, these findings suggest that mRNA synthesis and decay are not altered and do not account for differences in PLY protein levels. Thus our results are consistent with the hypothesis that changes in mRNA ΔG lead to changes in post-transcriptional processing and changes in protein abundance.

### ply mRNA ΔG affects host-pathogen interactions in vitro

**Fig 4:**
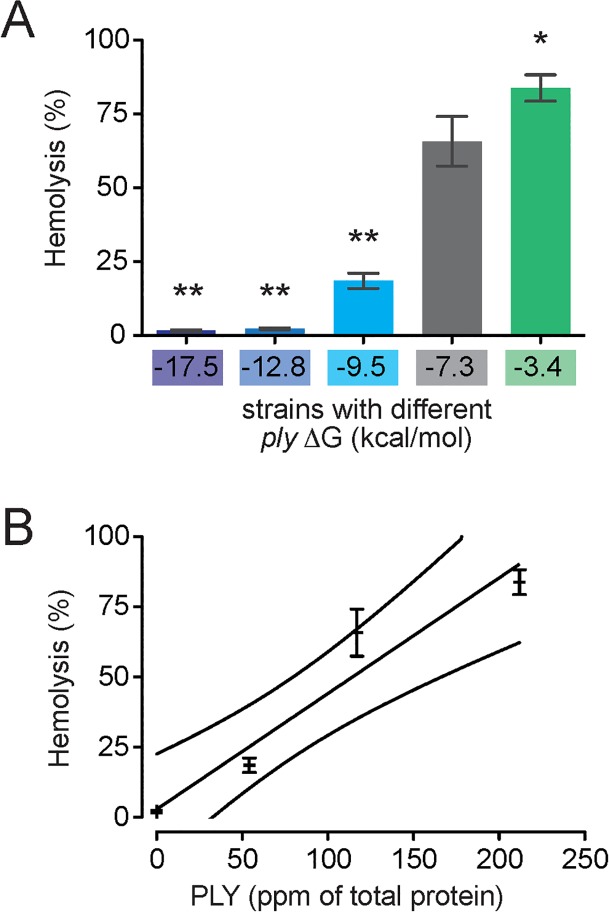
Differential PLY production by *S*. *pneumoniae* strains is directly related to PLY production. Lysis of primary human erythrocytes as assessed by hemoglobin release assay correlates with (A) *ply* 5’ mRNA ΔG and (B) PLY as measured by ELISA (R^2^ = 0.95; outside lines represent 95% CI). *, *P* < 0.05; **, *P* < 0.01 (ANOVA).

**Fig 5:**
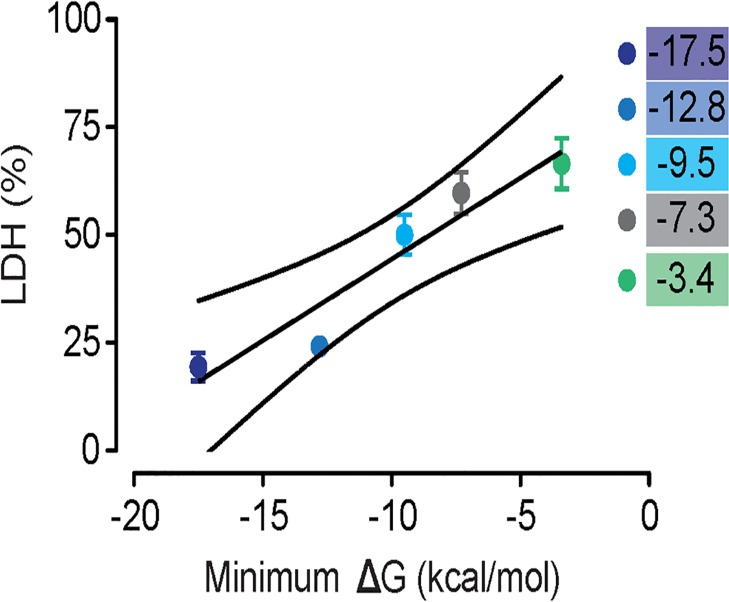
Lysis of human A549 respiratory epithelial cells as measured by LDH release correlates with predicted *ply* 5’ mRNA ΔG (R^2^ = 0.91; outside lines represent 95% CI; *P* < 0.0001, ANOVA for linear trend).

The previous experiments were performed using bacteria in culture. To determine whether altered 5’ mRNA ΔG also resulted in altered PLY production and cytotoxicity during infection of human cells, we examined PLY protein levels in two model systems: primary human erythrocytes and immortalized epithelial cells. PLY protein causes lysis of infected cells. We found that primary human erythrocytes exposed to *S*. *pneumoniae* strains exhibited lysis rates that correlated with predicted *ply* mRNA ΔG (Fig. 4A), suggesting that PLY protein levels expressed during infection correlate with predicted 5’ mRNA ΔG. We confirmed these differences in PLY protein levels by direct measurement using ELISA (Fig. 4B). In parallel, we found that measurement of A549 epithelial cell cytotoxicity demonstrated a similar positive correlation between *ply* mRNA ΔG and cellular damage (Fig. 5). Thus predicted differences in *ply* mRNA ΔG positively correlate with differences in lysis rates and differences in PLY protein expression levels during active infection of human tissue culture cells.

## Discussion

There is an emerging literature on the importance of 5’ mRNA folding ΔG to efficiency of translation and to overall levels of protein production [8,9,22–24]. Manipulation of codon bias, which may also impact protein levels, has been widely used as a method to enhance expression of genes in heterologous systems [5,25,26]. Rational alteration of 5’ mRNA ΔG has been suggested as a technique that might further aid such production [8,9]. Most studies of the ΔG/protein abundance relationship have focused on either synthetic constructs driving marker genes such as GFP [8,9] or on examination of naturally occurring coding sequences in model organisms [22,23,27,28]. In these studies, we rationally manipulated the predicted 5’ mRNA ΔG of a known pneumococcal virulence factor in order to determine concentration-dependent effects. Such investigations are not possible using traditional KO vs. WT approaches, and determining the relationship between protein abundance and virulence may drive more detailed understanding of pathogenic mechanisms.

Using unmarked, isogenic pneumococcal strains, we showed that rheostat-like control of PLY production is feasible and that changes in ΔG are sufficient to alter host-pathogen interactions. Alteration in expression did not depend on mRNA abundance or stability but occurred at the level of translation. As predicted, there was a direct correlation between PLY production and target cell lysis using simplified *in vitro* systems, and we provided proof-of-principle for the use of such systems to assist in the study of host-pathogen interactions. Expanded testing of modified strains in animal models of will allow assessment of the durability of altered PLY regulation during *S*. *pneumoniae* infection. Such models will facilitate understanding of the role of PLY expression levels at different stages and sites of infection.

Our findings are in agreement with those of Coleman et al., who used rational alteration of codon pair bias to alter PLY expression and found attenuation of inflammatory responses *in vivo* [7]. In contrast to that work, we alter PLY expression not by altering codon pair bias but by altering mRNA ΔG. Moreover, rather than altering the entire sequence, we were able to alter a short and specific part of the 5' region to alter PLY protein levels and lysis activity. The ability to alter a short portion of genetic sequence provides a potentially simple and straightforward tool for precisely regulating any bacterial protein involved in pathogenesis without having to alter large sections of the coding sequence and also without *a priori* knowledge of the host cell's codon preferences.

The correlation between 5’ mRNA ΔG and overall protein production is not perfectly linear, and other factors, including codon bias, GC content, and alteration of folding by other segments of the mRNA may have substantial effects on efficiency. Because of the limited number of strains investigated, we were unable to formally evaluate the role of 5’ mRNA ΔG while rigorously controlling each of these other variables, and, as noted above, our lowest expressing strains (ΔG = -17.5 and -12.8) had low 5’ CAI in addition to low 5’ mRNA ΔG. However, the other three strains (ΔG = -9.5, -7.3, and -3.4) did not differ substantially in 5’ CAI and had altered PLY production consistent with a role for 5’ mRNA ΔG. Whether this technique is widely applicable to alter protein production across a range of target genes and hosts must be determined experimentally. Despite these limitations, rational alteration of 5’ mRNA ΔG has potential as an important new tool in bacterial pathogenesis research. Given the increasing availability of whole genome sequences, much emphasis has been placed on the presence or absence of specific factors as markers of potential virulence in individual strains. Much less is known about the impact of relative expression levels on pathogenesis. The ability to investigate threshold and concentration-dependent effects of specific virulence factors without a requirement for non-native promoters, chemical inducers, or selective markers is extremely valuable and merits further exploration in a variety of systems.

## References

[1] Shockett PE, Schatz DG. Diverse strategies for tetracycline-regulated inducible gene expression. Proc Natl Acad Sci USA. 1996;93:5173–5176. 8643548 10.1073/pnas.93.11.5173 PMC39217

[2] Hershberg R, Petrov DA. Selection on codon bias. Annu Rev Genet. 2008;42:287–299. 10.1146/annurev.genet.42.110807.091442 18983258

[3] Ikemura T. Codon usage and tRNA content in unicellular and multicellular organisms. Mol Biol Evol. 1985;2:13–34. 3916708 10.1093/oxfordjournals.molbev.a040335

[4] Stoletzki N, Eyre-Walker A. Synonymous codon usage in Escherichia coli: selection for translational accuracy. Mol Biol Evol. 2007;24:374–381. 17101719 10.1093/molbev/msl166

[5] Sharp PM, Li WH. The codon Adaptation Index—a measure of directional synonymous codon usage bias, and its potential applications. Nucleic Acids Res. 1987;15:1281–1295. 3547335 10.1093/nar/15.3.1281 PMC340524

[6] Coleman JR, Papamichail D, Skiena S, Futcher B, Wimmer E, Mueller S. Virus attenuation by genome-scale changes in codon pair bias. Science. 2008;320:1784–1787. 10.1126/science.1155761 18583614 PMC2754401

[7] Coleman JR, Papamichail D, Yano M, García-Suárez MDM, Pirofski L-A. Designed reduction of Streptococcus pneumoniae pathogenicity via synthetic changes in virulence factor codon-pair bias. J Infect Dis. 2011;203:1264–1273. 10.1093/infdis/jir010 21343143 PMC3069727

[8] Kudla G, Murray AW, Tollervey D, Plotkin JB. Coding-sequence determinants of gene expression in Escherichia coli. Science. 2009;324:255–258. 10.1126/science.1170160 19359587 PMC3902468

[9] Goodman DB, Church GM, Kosuri S. Causes and effects of N-terminal codon bias in bacterial genes. Science. 2013;342:475–479. 10.1126/science.1241934 24072823

[10] Sung CK, Li H, Claverys JP, Morrison DA. An rpsL cassette, janus, for gene replacement through negative selection in Streptococcus pneumoniae. Appl Environ Microbiol. 2001;67:5190–5196. 11679344 10.1128/AEM.67.11.5190-5196.2001 PMC93289

[11] Berry AM, Yother J, Briles DE, Hansman D, Paton JC. Reduced virulence of a defined pneumolysin-negative mutant of Streptococcus pneumoniae. Infect Immun. 1989;57:2037–2042. 2731982 10.1128/iai.57.7.2037-2042.1989 PMC313838

[12] Los FCO, Randis TM, Aroian RV, Ratner AJ. Role of Pore-Forming Toxins in Bacterial Infectious Diseases. Microbiol Mol Biol Rev. 2013;77:173–207. 10.1128/MMBR.00052-12 23699254 PMC3668673

[13] Markham NR, Zuker M. DINAMelt web server for nucleic acid melting prediction. Nucleic Acids Res. 2005;33:W577–581. 15980540 10.1093/nar/gki591 PMC1160267

[14] Puigbò P, Bravo IG, Garcia-Vallve S. CAIcal: a combined set of tools to assess codon usage adaptation. Biol Direct. 2008;3:38 10.1186/1745-6150-3-38 18796141 PMC2553769

[15] Lacks S, Hotchkiss RD. A study of the genetic material determining an enzyme in Pneumococcus. Biochim Biophys Acta. 1960;39:508–518. 14413322 10.1016/0006-3002(60)90205-5

[16] Rampersaud R, Planet PJ, Randis TM, Kulkarni R, Aguilar JL, Lehrer RI, et al Inerolysin, a Cholesterol-Dependent Cytolysin Produced by Lactobacillus iners. J Bacteriol. 2011;193:1034–1041. 10.1128/JB.00694-10 21169489 PMC3067590

[17] Crook J, Tharpe JA, Johnson SE, Williams DB, Stinson AR, Facklam RR, et al Immunoreactivity of five monoclonal antibodies against the 37-kilodalton common cell wall protein (PsaA) of Streptococcus pneumoniae. Clin Diagn Lab Immunol. 1998;5:205–210. 9521144 10.1128/cdli.5.2.205-210.1998 PMC121359

[18] Johansson N, Kalin M, Giske CG, Hedlund J. Quantitative detection of Streptococcus pneumoniae from sputum samples with real-time quantitative polymerase chain reaction for etiologic diagnosis of community-acquired pneumonia. Diagn Microbiol Infect Dis. 2008;60:255–261. 18036762 10.1016/j.diagmicrobio.2007.10.011

[19] Gelber SE, Aguilar JL, Lewis KLT, Ratner AJ. Functional and phylogenetic characterization of Vaginolysin, the human-specific cytolysin from Gardnerella vaginalis. J Bacteriol. 2008;190:3896–3903. 10.1128/JB.01965-07 18390664 PMC2395025

[20] Aguilar JL, Kulkarni R, Randis TM, Soman S, Kikuchi A, Yin Y, et al Phosphatase-dependent regulation of epithelial mitogen-activated protein kinase responses to toxin-induced membrane pores. PLOS ONE. 2009;4:e8076 10.1371/journal.pone.0008076 19956644 PMC2778951

[21] Randis TM, Kulkarni R, Aguilar JL, Ratner AJ. Antibody-based detection and inhibition of vaginolysin, the Gardnerella vaginalis cytolysin. PLOS ONE. 2009;4:e5207 10.1371/journal.pone.0005207 19370149 PMC2666159

[22] Zur H, Tuller T. Strong association between mRNA folding strength and protein abundance in S. cerevisiae. EMBO Rep.; 2012;13:272–277. 10.1038/embor.2011.262 22249164 PMC3323128

[23] Shah P, Ding Y, Niemczyk M, Kudla G, Plotkin JB. Rate-limiting steps in yeast protein translation. Cell. 2013;153:1589–1601. 10.1016/j.cell.2013.05.049 23791185 PMC3694300

[24] Tuller T, Girshovich Y, Sella Y, Kreimer A, Freilich S, Kupiec M, et al Association between translation efficiency and horizontal gene transfer within microbial communities. Nucleic Acids Res. 2011;39:4743–4755. 10.1093/nar/gkr054 21343180 PMC3113575

[25] Supek F, Šmuc T. On relevance of codon usage to expression of synthetic and natural genes in Escherichia coli. Genetics. 2010;185:1129–1134. 10.1534/genetics.110.115477 20421604 PMC2900969

[26] Lee SF, Li Y-J, Halperin SA. Overcoming codon-usage bias in heterologous protein expression in Streptococcus gordonii. Microbiology. 2009;155:3581–3588. 10.1099/mic.0.030064-0 19696103

[27] Tuller T, Waldman YY, Kupiec M, Ruppin E. Translation efficiency is determined by both codon bias and folding energy. Proc Natl Acad Sci USA. 2010;107:3645–3650. 10.1073/pnas.0909910107 20133581 PMC2840511

[28] Roller M, Lucic V, Nagy I, Perica T, Vlahovicek K. Environmental shaping of codon usage and functional adaptation across microbial communities. Nucleic Acids Res. 2013;41:8842–8852. 10.1093/nar/gkt673 23921637 PMC3799439

